# Evolution of Microstructural and Mechanical Properties of Alloy 617B During Service on a Key-Component Test Platform at 700 °C

**DOI:** 10.3390/ma17215228

**Published:** 2024-10-27

**Authors:** Jiang Li, Xionghua Cui, Zheyi Yang, Liying Tang, Lei Zhang

**Affiliations:** Xi’an Thermal Power Research Institute Co., Ltd., Xi’an 710054, China; cuixionghua@tpri.com.cn (X.C.); yangzheyi@tpri.com.cn (Z.Y.); tangliying@tpri.com.cn (L.T.); zhanglei@tpri.com.cn (L.Z.)

**Keywords:** alloy 617B, long-term service, evolution of microstructural and mechanical properties, precipitation behavior, M_23_C_6_, *γ*′ phases, impact toughness

## Abstract

The evolution of the microstructural and mechanical properties of alloy 617B during long-term service on a key-component test platform at 700 °C was systematically investigated. The precipitation behavior and size changes of the M_23_C_6_ and *γ*′ phases were characterized by scanning electron microscopy (SEM) and transmission electron microscopy (TEM). The results showed that carbide M_23_C_6_ precipitated in the form of discontinuous particles, plates, or needles at grain boundaries and within grains, while the *γ*′ phase had a spherical shape and was distributed in a dispersed manner. With prolonged service time, both the M_23_C_6_ and *γ*′ phases gradually coarsened. After 24,000 h of service, the yield strength, tensile strength, and Brinell hardness of alloy 617B significantly increased; however, the impact toughness decreased, accompanied by intergranular embrittlement. The increase in precipitate volume fraction and its contribution to the strength of the alloy were evaluated by a precipitation strengthening model. The coarsening of M_23_C_6_ was identified as the main cause of embrittlement. The findings of this study provide important experimental data and theoretical support for the stability of 617B alloys under long-term high-temperature service conditions.

## 1. Introduction

To further improve the power generation efficiency of thermal power units, reduce coal consumption, and decrease greenhouse gas and other pollutant emissions, countries and regions such as Europe, Japan, the United States, and India have successively initiated research on 700 °C advanced ultra-supercritical (A-USC) power generation technology since the late 1990s [1,2]. This technology is characterized by a high working temperature and pressure, with a main steam temperature exceeding 700 °C and main steam pressure above 35 MPa in power generation units. As a result, power generation efficiency can be increased to 48%–50%, reducing coal consumption by approximately 40 g/kW·h, and significantly lowering emissions of greenhouse gases and harmful gases, such as CO_2_, SO_2_, and NO_x_ [3]. In 2015, China officially commissioned the first 700 °C key-component test platform on Unit 2 of the Huaneng Nanjing Power Plant, making it the first such platform established on an operational unit in the world [4].

As the steam parameters of power generation units increase, performance requirements for the building materials used in key components are becoming more stringent. Consequently, the selection of materials for high-temperature components remains a major bottleneck in the development of 700 °C A-USC power generation technology [5]. Traditional ferritic heat-resistant steels and austenitic heat-resistant steels, such as T92, TP347HFG, Super304H, and HR3C, can no longer meet the requirements of high-temperature heating surfaces. Therefore, the 700 °C key-component test platform was designed to test several high-temperature nickel-based alloys, including Inconel 740H, 617B, and Haynes 282 [6,7,8].

Among these, 617B is a solid-solution-strengthened nickel-based alloy derived from alloy 617 by incorporating a small amount of B elements and narrowly tolerated alloying elements. It also benefits from precipitation strengthening due to formation of the *γ*′ phase from trace amounts of elemental Al and Ti. Due to its excellent resistance to creep rupture, oxidation, corrosion, and flue gas corrosion, 617B has become a key focus of material research for 700 °C class power generation units worldwide [9,10,11,12,13]. Xiang et al. [9] found that primary precipitates during the aging process of 617B at 650–750 °C were M_23_C_6_ and *γ*′ phases, and these precipitates coarsened with an increase in time and temperature. Guo et al. [10] studied the precipitation behavior and mechanical properties of 617B during a 5000 h aging process at 750 °C and discovered that the increased strength and hardness of aged 617B were attributed to M_23_C_6_ and *γ*′ precipitates that formed at grain boundaries and within grains. Tytko et al. [11] showed that during the aging process of 617B at 700 °C, B elements were enriched at *γ*′/M_23_C_6_ and *γ*/M_23_C_6_ interfaces, as well as at grain boundaries. Wu et al. [12] studied the microstructural evolution of alloy 617 after aging from 482 °C to 871 °C, and found that the primary precipitates were M_23_C_6_, *γ*′, M_6_C, and TiN. Hou et al. [13] conducted an aging process on alloy 617B at 780 °C, as well as a corrosion test in a simulated coal-fired environment at the same temperature. The results showed that the *γ*′ phase did not appear before aging but grew to around 130 nm after 5000 h of aging.

Although the evolution of microstructure and the performance of alloy 617B have been reported [7,8,9,10,11,12,13], most of these studies utilized short testing durations that typically did not exceed 5000 h, making it difficult to accurately assess the stability of the microstructure and mechanical properties of 617B during long-term service [7,8,9]. In addition, many reported aging treatments were conducted at temperatures significantly higher than the actual operating temperatures of 617B to accelerate material aging [10,11], rendering the reported data of little practical use for engineering applications.

For 617B, a key candidate material for 700 °C class A-USC power generation units with high temperature and pressure, the evolution of its microstructure and mechanical properties during long-term service directly impacts the operational safety of the units. Because 617B has been used in super-heater tubes of 700 °C key-component test platforms, this study compared two groups of specimens from super-heater tubes (after 10,000 h or 24,000 h of service) with one group of specimens obtained from as-received tubes, to comprehensively investigate the evolution of the microstructural and mechanical properties evolution of this alloy during long-term service.

## 2. Materials and Methods

The 617B tubes used in the super-heater of the 700 °C key-component test platform were provided by Salzgitter Mannesmann Stainless Tubes GmbH (Remscheid, Germany), and measured 44.5 mm in diameter and 10 mm in wall thickness. Table 1 reflects the nominal chemical composition of 617B offered by the supplier.

Real-time temperature monitoring showed that the tube sections for tube specimen collection had a wall temperature between 679 °C and 685 °C, with an inner steam pressure of approximately 24.9 MPa. Tube specimens from the super-heater were collected twice during unit shutdowns; first after 10,000 h of service and then after 24,000 h. The as-received tube specimens (denoted as 0 h specimens) were used as controls. Room-temperature tensile tests were conducted according to the ASTM E8/E8M-2024 standard [14], and high-temperature tensile tests at 700 °C were conducted according to the ASTM E21-2020 [15] standard. All tensile tests were performed using an ETM305D-EX tensile testing machine (WANCE Test Equipment Co., Ltd., Shenzhen, China). Standard cylindrical specimens were used, featuring a gauge length of 24 mm and a diameter of 6 mm. Room temperature impact tests were conducted according to the ASTM E23-2023 standard [16], using Charpy V-notch impact specimens with dimensions of 10 × 7.5 × 10 mm on an RKP450 impact testing machine (ZwickRoell Testing Technology Co., Ltd., Ulm, Germany). Brinell hardness tests were conducted according to the ASTM E10-2023 standard, using an HB-3000C Brinell hardness tester (WANCE Test Equipment Co., Ltd., Shenzhen, China). To ensure accuracy, six parallel specimens were used for both room-temperature impact tests and Brinell hardness tests under each condition, while four parallel specimens were employed for tensile tests at both room and high temperatures. Fracture characteristics, microstructure, precipitate types, and distributions were characterized by a ZEISS LSM 700 metallographic microscope (Jena, Germany), Thermo Fisher Apreo S scanning electron microscope (SEM) (Waltham, MA, USA), and JEM-3010 transmission electron microscope (TEM) (JEOL, Tokyo, Japan). SEM samples were prepared by performing 2.5 µm diamond polishing following conventional mechanical polishing to achieve a mirror-like finish. To etch the polished surfaces, Aqua regia (a mixture of 3 parts HCl and 1 part HNO_3_) was used for the as-received sample. TEM samples were prepared by cutting 3 mm diameter discs from the bulk material and mechanically polishing them to a thickness of approximately 100 µm. The discs were then electropolished using an MTP-1A double-jet system(Shanghai Jiao Da electromechanical technology development Co., Ltd., Shanghai, China) at −30 °C with 35 V in a solution of 90% ethanol and 10% perchloric acid.

## 3. Results

### 3.1. Impact Properties

**Figure 1:**
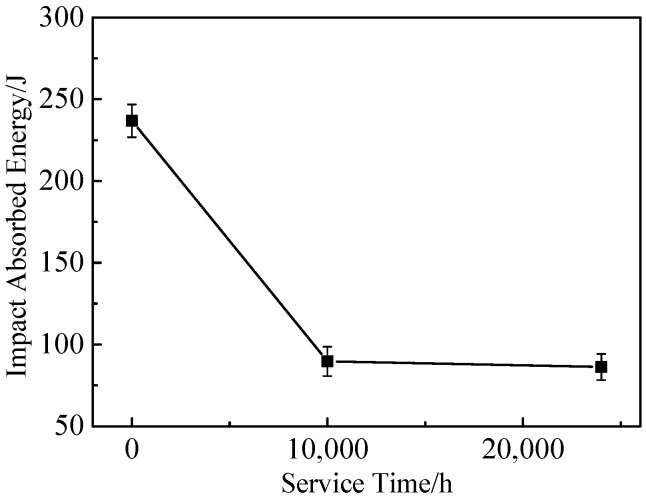
Variations in impact energy absorption of 617B at room temperature with service time.

Figure 1 shows the impact energy absorption trend of 617B at room temperature as a function of service time. The impact energy values were converted to the corresponding values for the 10 mm wide specimens in accordance with the EN 10216-2:2016 standard [17] As shown in Figure 1, the impact energy absorption of 617B significantly decreased during the first 10,000 h of service, with its value dropping to 89.9 J after 10,000 h, which was a reduction of 62.1% compared to the 0 h value. Subsequently, the impact energy stabilized, reaching 86.3 J after 24,000 h of service.

**Figure 2:**
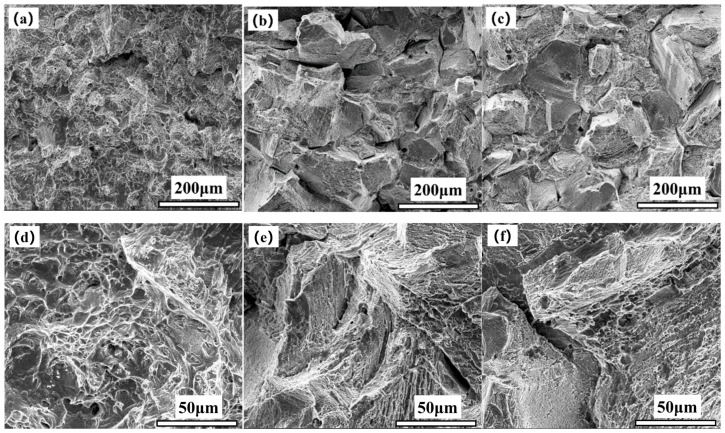
Macroscopic fracture morphology of the crack propagation region of 617B impact specimens at room temperature: (**a**,**d**) 0 h, (**b**,**e**) 10,000 h, and (**c**,**f**) 24,000 h.

Figure 2 show the fracture morphology of the 617B crack propagation region after impact testing. As shown in Figure 2a,d, at 0 h, the fracture surface of the crack propagation region contained numerous dimples with varying depths and sizes, with no straight grain boundaries, suggesting typical ductile fracture. After 10,000 h of service, the fracture showed a trend of intergranular fracture (Figure 2b), with small dimples and numerous secondary cracks visible on the grain surface at high magnification (Figure 2e). After 24,000 h of service, intergranular fracture became more pronounced (Figure 2c), and small dimples and numerous secondary cracks were still observed on the grain surface at high magnification (Figure 2f).

### 3.2. Tensile Properties

**Figure 3:**
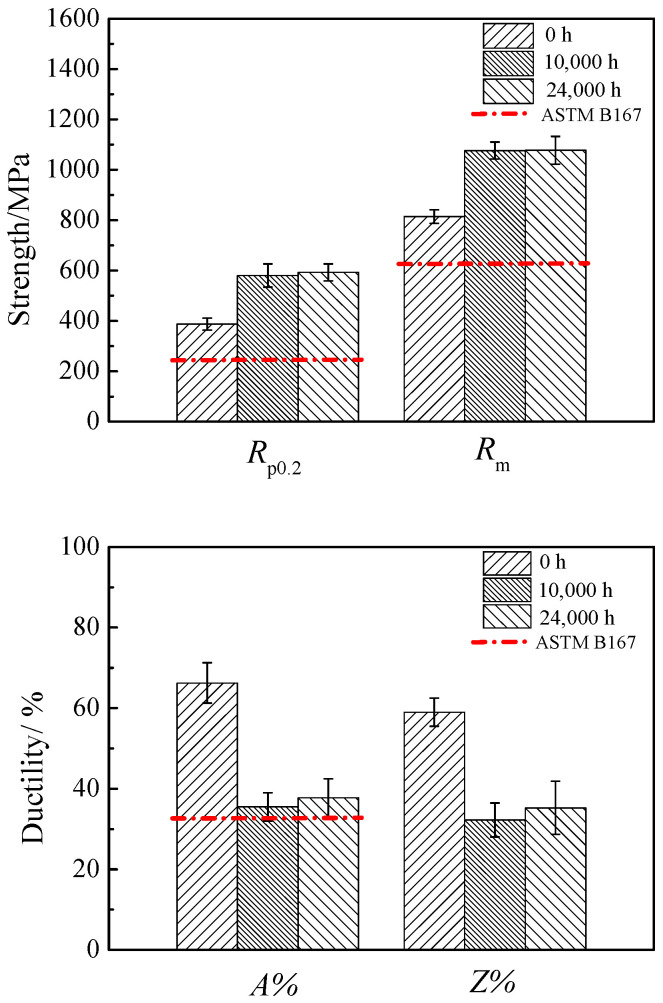
Variations in tensile properties of 617B at room temperature with service time.

**Figure 4:**
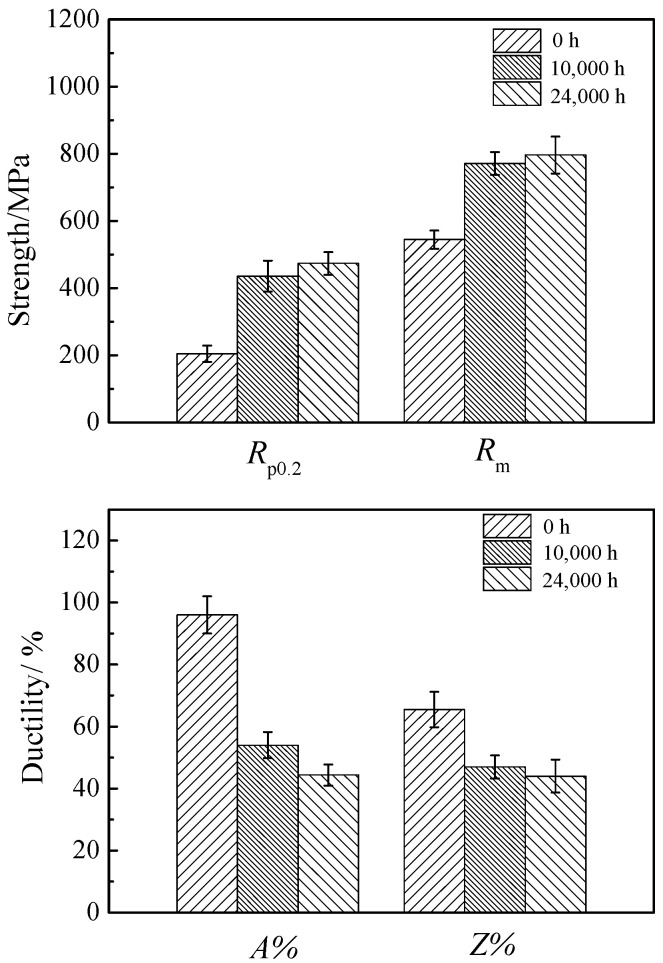
Variations in the tensile properties of 617B at 700 °C with service time.

Figure 3 and Figure 4 present the changes in tensile properties of 617B at room temperature and 700 °C as a function of service time. The yield strength (*R*_p0.2_), tensile strength (*R*_m_), and elongation (*A*) at room temperature for all three specimens met the requirements of the ASTM B167-2021 standard [18]. With an increase in service time, both *R*_p0.2_ and *R*_m_ at room temperature and 700 °C showed an upward trend. Compared to the as-received condition, *R*_p0.2_ and *R*_m_ at room temperature increased by 52.9% and 32.3% after 24,000 h of service, respectively, while *R*_p0.2_ and *R*_m_ at 700 °C increased by 132% and 42.2%, respectively. However, *A* and reduction of area (*Z*) decreased with prolonged service time. Compared to the as-received condition, *A* and *Z* at room temperature decreased by 43% and 40.3% after 24,000 h of service, respectively, while *A* and *Z* at 700 °C decreased by 54.2% and 32.8%, respectively. Despite a reduction in ductility, the *A* of 617B after 24,000 h of service met ASTM B167-2021 requirements by remaining ≥30%, indicating that the alloy retained good ductility even after long-term service.

**Figure 5:**
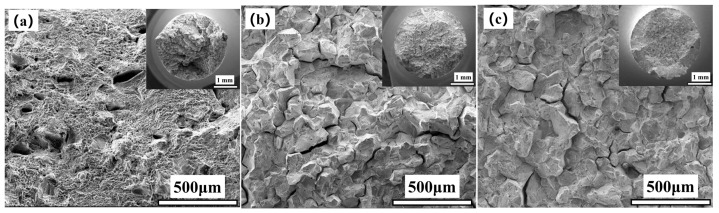
Fracture morphology of 617B tensile specimens at room temperature: (**a**) 0 h, (**b**) 10,000 h, and (**c**) 24,000 h.

**Figure 6:**
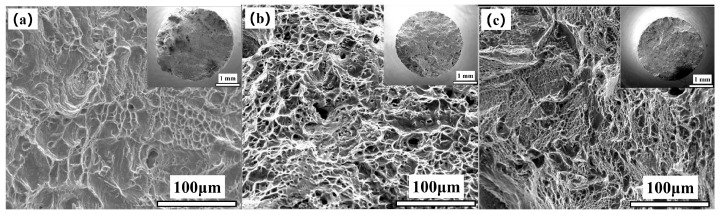
Fracture morphology of 617B tensile specimens at 700 °C: (**a**) 0 h, (**b**) 10,000 h, and (**c**) 24,000 h.

Figure 5 and Figure 6 present the fracture morphology of 617B tensile specimens at room temperature and 700 °C after different service times. As shown in Figure 5, (1) at room temperature, the 0 h specimens exhibited an uneven macroscopic fracture morphology, with microscopic fracture morphology typical of ductile fracture. (2) After 10,000 h of service, macroscopic fracture morphology showed no significant necking, with a granular appearance on the fractured cross-section and shear lips at the edges, while the central region of the fracture showed characteristics of intergranular fracture, alongside secondary intergranular cracks. (3) Fracture morphology after 24,000 h of service was similar to that after 10,000 h. In high-temperature tensile tests (Figure 6), the macroscopic fracture morphology of all three groups of specimens was uneven, and the microscopic fracture morphology demonstrated characteristics of ductile fracture with numerous dimples.

### 3.3. Brinell Hardness

**Figure 7:**
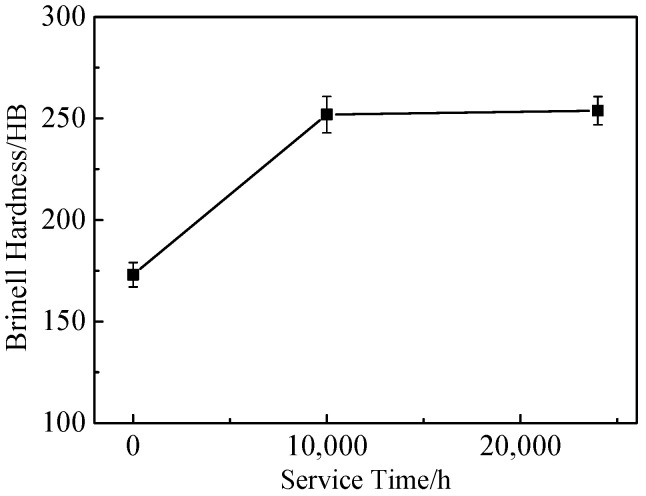
Variations in Brinell hardness of 617B with service time.

Figure 7 shows the changes in Brinell hardness of 617B with service time. Brinell hardness increased with prolonged service time, which was similar to the trend observed for tensile strength at room temperature. After 24,000 h of service, Brinell hardness increased by approximately 46.7% compared to the as-received condition.

### 3.4. Microstructural Analysis

**Figure 8:**
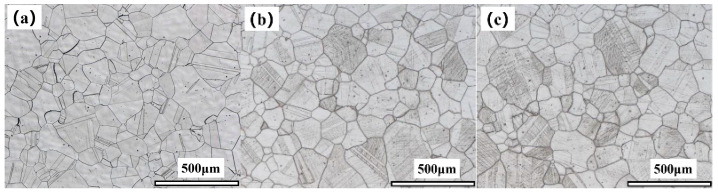
Metallographic structures of 617B after different service times: (**a**) 0 h, (**b**) 10,000 h, and (**c**) 24,000 h.

Figure 8 presents the metallographic structures of the three groups of 617B specimens. These specimens exhibited a typical equiaxed austenitic structure, with a few twins inside the grains and a grain size corresponding to grades 2–3. No significant changes in grain size were observed with increased service time, although the number of precipitates at grain boundaries and within grains gradually increased.

**Figure 9:**
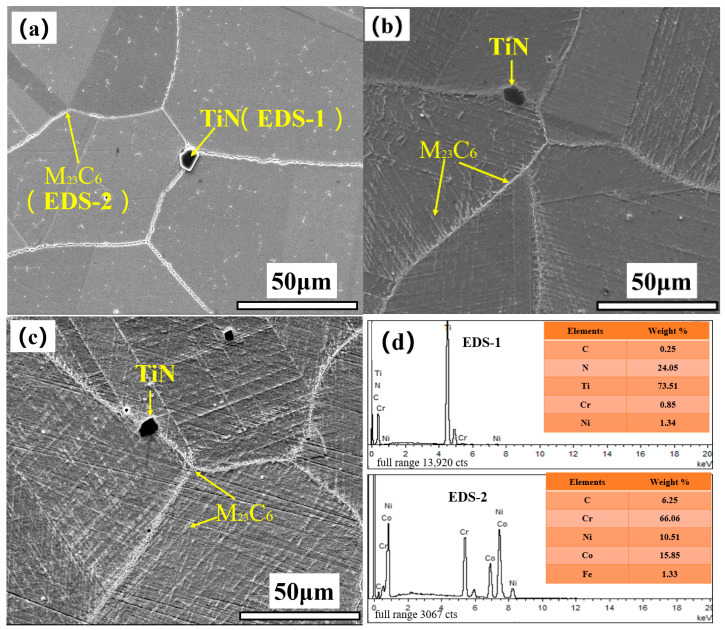

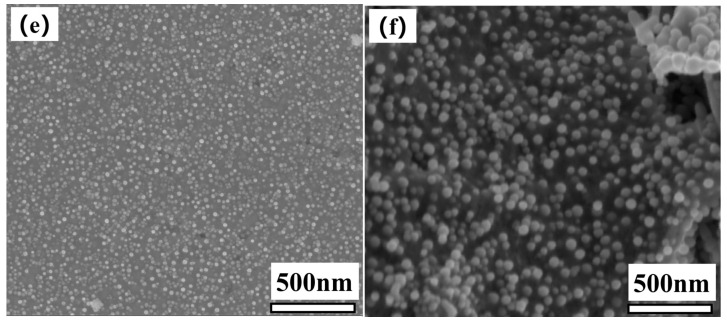
SEM morphology and EDS analysis of 617B: (**a**) 0 h, (**b**) 10,000 h, (**c**) 24,000 h; (**d**) EDS analysis, (**e**) *γ*′ phase within grains after 10,000 h, and (**f**) *γ*′ phase within grains after 24,000 h.

**Figure 10:**
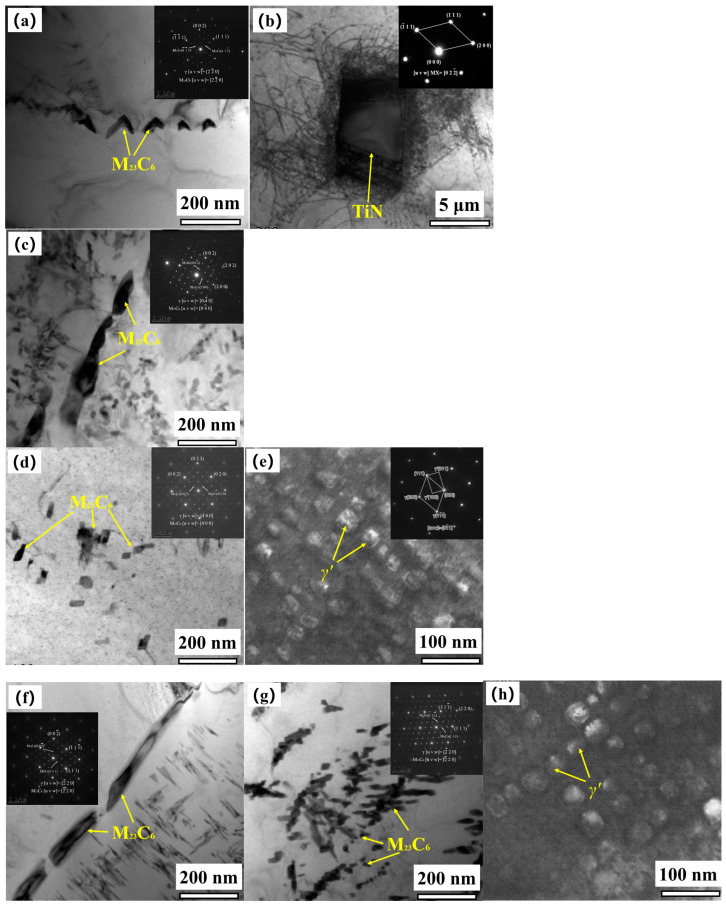
TEM morphology and electron diffraction patterns of 617B: (**a**,**b**) 0 h, (**c**–**e**) 10,000 h, and (**f**–**h**) 24,000 h.

Figure 9 shows the SEM microstructure and EDS analysis results of the three groups of 617B specimens, and Figure 10 presents the TEM images and selected area electron diffraction (SAED) patterns.

As shown in Figure 9a, block-like precipitates were distributed near grain boundaries of the as-received alloy, with an approximate size of 6 μm. EDS analysis revealed that these precipitates were rich in Ti and N (Figure 9d). TEM and SAED analyses (Figure 10b) further confirmed that these precipitates were undissolved primary TiN, with a face-centered cubic structure. Notably, the quantity and distribution of these precipitates did not change significantly during service (Figure 9b,c). Additionally, discontinuous white particles were distributed along grain boundaries in the as-received alloy (Figure 9a), and EDS analysis showed that these particles were rich in Cr (Figure 9d). SAED analysis indicated that these particles comprised carbide M_23_C_6_ (Figure 10a), with a lattice constant three times that of the matrix, resulting in diffraction spots spaced at one-third intervals compared to those of the matrix. M_23_C_6_ at grain boundaries of the as-received alloy measured approximately 27–69 nm in length, with no M_23_C_6_ or *γ*′ phase observed inside the grains.

After 10,000 h of service, the SEM images revealed a significant increase in the precipitation of M_23_C_6_ within grains; no significant changes in M_23_C_6_ at grain boundaries were observed (Figure 9b). The *γ*′ phase had a spherical shape and was distributed in a dispersed manner within grains, averaging approximately 17–25 nm in size (Figure 9e). TEM images showed that M_23_C_6_ at grain boundaries visibly coarsened, with lengths reaching 155–340 nm, and these particles were distributed discontinuously (Figure 10c). Additionally, plate-like or needle-like M_23_C_6_ particles were observed within grains (Figure 10d), with lengths ranging from about 34 to 120 nm. Under dark-field imaging, the *γ*′ phase, which was dispersed within grains, was found to be coherent with the matrix (Figure 10e).

After 24,000 h of service, SEM images showed a further increase in the precipitation of M_23_C_6_ at grain boundaries and within grains (Figure 9c). M_23_C_6_ within grains exhibited directional precipitation, and the size of the *γ*′ phase, which was dispersed within grains, slightly increased, with an average size of approximately 42–66 nm (Figure 9f). TEM analysis indicated that the M_23_C_6_ phase at grain boundaries, which remained discontinuous and was not fully interconnected despite its coarsening trend, had significantly increased in size, ranging from about 206 to 445 nm in length (Figure 10f). Within grains, the growth of plate-like or needle-like M_23_C_6_ preferentially occurred along certain orientations, with an observable trend toward clustering, and the length of this carbide phase significantly increased after service, reaching from 51 to 240 nm (Figure 10g). The size of the *γ*′ phase, which was dispersed within grains, also slightly increased (Figure 10h). Throughout the service period, no σ phase precipitation was observed.

## 4. Discussion

The results of this study indicated that the main precipitates in 617B alloy after long-term service consisted of M_23_C_6_ and *γ*′ phases, with (1) M_23_C_6_ precipitates located at both grain boundaries and within grains. At grain boundaries, M_23_C_6_ was distributed as discontinuous particles, while M_23_C_6_ within grains exhibited a plate-like or needle-like morphology. (2) The *γ*′ phase was dispersed within grains in a spherical shape, and (3) as service time increased, both the M_23_C_6_ and *γ*′ phases gradually coarsened (Figure 9 and Figure 10). The sizes of the M_23_C_6_ and *γ*′ precipitates at different service times are listed in Table 2, which were slightly smaller than those reported for the same types of precipitates in other studies [10,13]. This discrepancy was primarily attributed to the fact that the aging temperatures in the literature were significantly higher than the actual service temperature of 617B in this study.

A similar study with a 10,000 h exposure also reported the presence of M_23_C_6_ and *γ*′ phases, along with a detailed analysis of elemental redistribution during aging [19]. In that study, a more pronounced coarsening of M_23_C_6_ precipitates was observed, which can likely be attributed to the higher aging temperatures compared to the lower service temperature used in this study.

In the 700 °C service environment, 617B maintained good mechanical properties even after 24,000 h of service. Compared to the as-received condition, the *R*_p0.2_ and *R*_m_ values at room temperature and 700 °C significantly increased after service (Figure 3 and Figure 4), with the Brinell hardness values also showing an upward trend (Figure 7). This increase in strength and hardness was primarily related to the size and distribution of the precipitates [9,10,11,13]. According to the precipitation strengthening theory [20,21,22]:

Δ*τ* = *αGb*/*λ* (1)

where Δ*τ* represents an increase in strength due to precipitates; *α* is a constant; *G* is the shear modulus; *b* denotes the Burgers vector of the dislocations; and *λ* is the average spacing between precipitates. In this equation, *λ* was inversely proportional to the average size (*d*) and volume fraction (*f*) of the precipitates. The results showed (1) that the M_23_C_6_ phase at grain boundaries and within grains, as well as the *γ*′ phase within grains, coarsened with increasing service time (Table 2). (2) The *f* of M_23_C_6_, as well as the *f* of the *γ*′ phase within grains, also significantly increased with service time (Figure 8). These two types of changes with prolonged service time jointly led to a reduction in *λ*, thus, resulting in an increase in *Δτ*, which explained the increase in strength after service. Additionally, the newly formed *γ*′ precipitates during service were small in size and remained coherent with the matrix, effectively impeding dislocation movement [12]. The M_23_C_6_ phase within grains, which differed from the matrix in composition and structure, generated interfacial energy at phase boundaries, obstructing dislocation slip, and enhancing alloy strength and hardness [23].

However, the impact toughness of 617B decreased after long-term service. After 10,000 h of service, the impact energy absorption decreased to 89.9 J, which was a reduction of 62.1% compared to the as-received condition (Figure 1). The elongation and reduction in area at room temperature and 700 °C also showed a downward trend as the service time increased (Figure 3 and Figure 4). The material exhibited signs of embrittlement after service, with both the impact fracture and room-temperature tensile fracture showing intergranular fracture characteristics, which were related to the precipitation of M_23_C_6_ at grain boundaries [10,24]. In the as-received condition, the quantity of M_23_C_6_ precipitates at grain boundaries was small, with sizes ranging from 42 to 85 nm, and grain boundary cohesion was relatively high. After service, as the size and quantity of M_23_C_6_ precipitates increased (Table 2), cohesion at grain boundaries decreased, causing the grain boundaries to become crack initiation sites during deformation [25].

No σ phase precipitation, as reported in the literature, was observed in 617B during long-term service. This was primarily because the aging temperature in the literature [12] was 750 °C, which was significantly higher than the actual service temperature of 617B.

## 5. Conclusions

1. The main precipitates during the long-term service of 617B were M_23_C_6_ and *γ*′ phases. M_23_C_6_ precipitates along grain boundaries and within grains consisted of discontinuous particles, plates, or needles, while the *γ*′ phase had a spherical shape and was distributed in a dispersed manner within grains. Both the M_23_C_6_ and *γ*′ phases coarsened with prolonged service time.
2. The yield strength and tensile strength of 617B increased with service time in both room-temperature and 700 °C tensile tests. After 24,000 h of service, the room-temperature tensile yield strength, tensile strength, and elongation met the standard requirements, indicating good mechanical stability. This was primarily attributed to the precipitation strengthening effect of the M_23_C_6_ and *γ*′ phases.
3. The impact energy absorption of 617B at room temperature decreased significantly after 10,000 h of service, followed by a trend toward stabilization. This reduction was mainly due to coarsening of the M_23_C_6_ phase at grain boundaries, which reduced grain boundary cohesion.

## Figures and Tables

**Table 1:** Chemical compositions of the 617B alloy (wt. %).

Element	617B	Element	617B	Element	617B
C	0.059	Cu	0.041	Co	12.06
Si	0.057	Mo	8.68	Fe	1.32
Mn	0.046	Ti	0.45	H	0.00018
P	<0.005	Al	0.45	N	0.010
S	0.0007	As	<0.005	O	0.0004
Cr	21.96	B	<0.005	Pb	<0.00001
Ni	54.21	Bi	<0.00001	Sb	<0.00001

**Table 2:** Precipitate size statistics in 617B during service.

Service Time (h)	Length of the Grain Boundary M_23_C_6_ (nm)	Length of Intragranular M_23_C_6_ (nm)	Intragranular γ′ Phase (nm)
0	27–69	/	/
10,000	155–340	34–120	17–25
24,000	206–445	51–240	42–66

## Data Availability

The original contributions presented in the study are included in the article, further inquiries can be directed to the corresponding author.
